# Cigarette smoke modulates virulence-related properties and host immune responses in *Klebsiella pneumoniae*

**DOI:** 10.3389/fcimb.2026.1787424

**Published:** 2026-07-23

**Authors:** Hala O. Eltwisy, Medhat Abdel-Fattah, Khaled N. M. Elsayed, Rasha Assad Assiri, Salwa Seif Eldin, Amal A. Elkhawaga, Marwa M. Khalifa, Mohamed Ahmed

**Affiliations:** 1 Department of Botany and Microbiology, Faculty of Science, Beni-Suef University, Beni-Suef, Egypt; 2 Biology Department, Faculty of Education and Arts, Sohar University, Sohar, Oman; 3 Department of Basic Medical Sciences, College of Medicine, Princess Nourah bint Abdulrahman University, Riyadh, Saudi Arabia; 4 King Saud University Celiac Disease Research Chair, Department of Pediatric, college of medicine King Saud University, Riyadh, Saudi Arabia; 5 Department of Medical Microbiology and Immunology, Faculty of Medicine, Assiut University, Assiut, Egypt; 6 Department of Microbiology and Immunology, Faculty of Pharmacy, Assiut University, Assiut, Egypt; 7 College of Medicine, Dhofar University, Salalah, Oman

**Keywords:** cigarette smoke extract, host-pathogen interaction, immune dysregulation, intracellular invasion, *Klebsiella pneumoniae*, THP-1 macrophages

## Abstract

**Introduction:**

Cigarette smoke is a major risk factor for respiratory infections and chronic inflammatory lung diseases, yet the mechanisms through which it alters host–pathogen interactions remain incompletely understood. *Klebsiella pneumoniae* is an opportunistic Gram-negative pathogen that causes severe infections, particularly in susceptible individuals. This study investigated the effects of cigarette smoke extract (CSE) on *K. pneumoniae* virulence-associated traits and host immune responses.

**Methods:**

Three multidrug-resistant, extended-spectrum β-lactamase-producing clinical *K. pneumoniae* isolates were exposed to CSE. Bacterial growth, virulence gene expression (*mrkA, luxS*, and *AcrB*), biofilm formation, antimicrobial susceptibility, macrophage invasion, neutrophil phagocytosis, and cytokine responses in THP-1-derived macrophages were evaluated using microbiological, molecular, and cell culture assays.

**Results:**

High concentrations of CSE (≥25%) suppressed bacterial growth, whereas 5–10% CSE had minimal effects. Exposure to 10% CSE significantly upregulated *mrkA, luxS*, and *AcrB* expression. Biofilm formation increased by 20.8–37.0%, and minimum inhibitory concentrations of ciprofloxacin and tigecycline increased two-fold following CSE exposure. CSE enhanced bacterial invasion of THP-1 macrophages and impaired neutrophil phagocytosis, reducing bacterial uptake by 17.8% and 29.9% after 30 and 60 min, respectively. Furthermore, CSE promoted a pro-inflammatory response characterized by increased TNF-α and IL-1β expression and reduced IL-10 expression across multiple time points.

**Discussion:**

CSE enhances the virulence potential of *K. pneumoniae* while disrupting innate immune defenses and immune regulation. These findings demonstrate a harmful synergy between cigarette smoke and bacterial infection that may contribute to the increased susceptibility and severity of respiratory infections observed in smokers.

## 1 Introduction

Cigarette smoking (CS) is a leading cause of preventable disease and death worldwide, strongly associated with chronic respiratory conditions such as chronic obstructive pulmonary disease (COPD), increased susceptibility to pulmonary infections, and impaired immune function. CS is a global public health issue with about 5 million deaths each year (Qin et al., 2020). The World Health Organization (WHO) has warned that in the decade 2020–2030 tobacco will kill 10 million people per year, 70% of which occur in developing countries (Palallo et al., 2019). CS is a risk factor for cardiovascular disease and is responsible for 20% of CVD deaths in the United States (Duncan et al., 2019). In 2004 an estimated 600,000 people worldwide died of diseases caused by prolonged exposure to secondhand smoke (SHS). About 28% of these deaths occurred in children under the age of 15 (Kuntz and Lampert, 2016). CS is a risk factor for premature death and several chronic diseases (Axelrod et al., 2021).

Cigarette smoke contains thousands of toxic compounds, including reactive oxygen species (ROS), aldehydes, and carbonyl-modified proteins, which collectively impair immune cell function. Alveolar macrophages are particularly affected by cigarette smoke, exhibiting impaired phagocytic capacity, dysregulated cytokine production, and altered signaling pathways (Lugg et al., 2022).

Smokers are deficient in cilia with abnormal structures and functions of ciliated cells. Long-term smoking can lead to an increase in the number of abnormal cilia in the bronchi and may damage the tracheobronchial function (Cao et al., 2020). Chronic inhalation of cigarette smoke alters cell proliferation, endothelial function, and immune response (Berkowitz et al., 2018). CS effects include: loss of phagocytic capacity, failure to contain the intracellular growth of mycobacteria in macrophages, attenuation of cytokine and chemokine response as well as attenuation of apoptosis (Gomez et al., 2020). CSE was shown to promote neutrophil degranulation, which may cause an exaggerated response to invading bacteria and induce local tissue damage (White et al., 2018). Tobacco smoke exposure is related to increased colonisation by pathogenic bacteria in middle nasal meatus and elevation of IgA and IgG in peripheral blood (Bugova et al., 2018). Cigarette smoking produces factors which increase bacterial growth and biofilm formation in the bronchoalveolar lavage (Vargas Buonfiglio et al., 2018).

Among the numerous pathogens associated with respiratory disease, *Klebsiella pneumoniae* (*K. pneumoniae*) stands out as a clinically significant Gram-negative bacterium capable of causing severe pneumonia, particularly in hospitalized patients, immunocompromised individuals and smokers (Caneiras et al., 2019). *K. pneumoniae*-induced pneumonia has been one of the most urgent global health threats due to its high incidence of complications, and an approximately 50% mortality rate under current antimicrobial therapy (Lou et al., 2018). Antibiotic resistance is an increasingly urgent problem, predicted to cause 10 million annual deaths worldwide by the year 2050. *Klebsiella* spp., including *K. pneumoniae*, are responsible for ~10% of nosocomial infections in the United States (Band et al., 2018). All *K. pneumoniae* isolates from smokers of inpatients were highly resistance to most antimicrobials as compare with those isolates from non-smokers (Aljanaby et al., 2018).

The high incidence of *K. pneumoniae* infections is favoured by the ability of this pathogen to colonize the gastrointestinal (GI) tract, which is associated with subsequent infection (Gato et al., 2020). The formation of biofilms on respiratory devices can lead to pneumonia via bacterial dissemination in the lower respiratory tract, with *K. pneumoniae* being responsible for 9.8% of ventilator-associated pneumonia (VAP) cases in intensive care units (Guilhen et al., 2019). The exposure of electric and tobacco cigar smoke inhibited the growth of *K. pneumoniae* colonies. The electric cigar smoke showed higher inhibition against bacteria (Muhamad Ramdan et al., 2020).

Despite the well-established link between cigarette smoke and increased risk of respiratory infections, the underlying mechanisms by which cigarette smoke modulates host-pathogen interactions remain incompletely understood. The present study aimed to investigate the dual impact of cigarette smoke extract (CSE) on both bacterial virulence and host immune responses. Specifically, we examined how CSE affects the growth, virulence gene expression (*mrkA*, *luxS*, *AcrB*), biofilm formation, and antimicrobial susceptibility of *K. pneumoniae*. In addition, we evaluated its ability to survive within macrophages and assessed neutrophil phagocytic activity as a measure of innate immune function. In parallel, we analyzed the cytokine response of THP-1–derived macrophages during infection, including both pro-inflammatory (TNF-α, IL-1β) and anti-inflammatory (IL-10) mediators, and extended this analysis across multiple time points to confirm the consistency of the response. Understanding these interactions is essential for elucidating the mechanisms by which cigarette smoke enhances bacterial virulence potential and disrupts host immunity, and may guide future therapeutic strategies for smoke-exposed individuals.

## 2 Methods

### 2.1 Cigarette smoke extract preparation

A 60-ml syringe (BD) containing 10 ml Tryptic soy broth was connected to a Marlboro cigarette (Philip Morris International Inc., New York, NY), and 50 ml smoke was aspirated, agitated for 15 s, and expelled. This process was repeated 10 times and the CSE solution was sterilized through a 0.22-μm filter, and its pH was adjusted to 7.4. The optical density of the CSE solution was checked by measuring the absorbance at 320 nm using a microplate Spectrophotometer (EPOCH, USA), which showed little difference between different preparations. This CSE solution was considered 100% CSE (Lacoma et al., 2019; Rodriguez-Fernandez et al., 2021).

### 2.2 Bacterial isolates and antimicrobial susceptibility testing

Three clinical isolates of *Klebsiella pneumoniae* (designated K1, K2, and K3) were obtained from patients with pneumonia. Isolates were selected based on their multidrug-resistant (MDR) phenotype and were used to evaluate the impact of cigarette smoke extract (CSE) on bacterial virulence and host-pathogen interactions. Bacterial identification was confirmed using standard microbiological methods. Antimicrobial susceptibility testing was performed using the VITEK 2 system (bioMérieux, France) and further validated by the disk diffusion method in accordance with Clinical and Laboratory Standards Institute (CLSI) guidelines.

A comprehensive panel of antibiotics representing multiple antimicrobial classes was tested, including β-lactam/β-lactamase inhibitor combinations (amoxicillin–clavulanic acid, piperacillin–tazobactam), cephalosporins (ceftazidime, ceftriaxone, cefepime), carbapenems (ertapenem, imipenem, meropenem), aminoglycosides (amikacin, gentamicin), fluoroquinolones (ciprofloxacin, levofloxacin), folate pathway inhibitors (trimethoprim–sulfamethoxazole), as well as nitrofurantoin, fosfomycin, colistin, and tigecycline.

Quality control for antimicrobial susceptibility testing and MIC determination was performed using *Escherichia coli* ATCC 25922 in accordance with CLSI guidelines, and all results were within the recommended reference ranges.

All isolates were classified as MDR based on resistance to at least three antimicrobial classes. In addition, the isolates exhibited an extended-spectrum β-lactamase (ESBL) phenotype, as indicated by resistance to third- and fourth-generation cephalosporins. The detailed antimicrobial susceptibility profiles of the isolates are presented in **Table 1**.

**Table 1:** Antimicrobial susceptibility profile of *K. pneumoniae* clinical isolates.

Antibiotics class	Antibiotic	K1	K2	K3
β-lactam/β-lactamase inhibitor	Amoxicillin–clavulanic acid	R	R	R
	Piperacillin–tazobactam	R	I	R
Cephalosporins (3rd/4th gen)	Ceftazidime	R	R	R
	Ceftriaxone	R	R	R
	Cefepime	R	R	R
Carbapenems	Ertapenem	R	I	R
	Imipenem	S	S	S
	Meropenem	S	S	S
Aminoglycosides	Amikacin	S	S	R
	Gentamicin	S	R	R
Fluoroquinolones	Ciprofloxacin	R	S	R
	Levofloxacin	R	R	R
Folate pathway inhibitors	Trimethoprim–sulfamethoxazole	R	R	R
Nitrofuran	Nitrofurantoin	S	I	S
Phosphonic acid derivative	Fosfomycin	S	S	S
Polymyxins	Colistin	S	S	S
Glycylcyclines	Tigecycline	S	S	S

In preparation for the experiments, *K. pneumoniae* strains were streaked onto MacConkey agar (Oxoid; Thermo Fisher Oxoid, UK), incubated for 18 h at 37 °C, and single colonies transferred to 3 mL of tryptic soy broth (TSB; Thermo Fisher Oxoid, UK) in 15 mL plastic sterile tubes and propagated in a shaking incubator at 180 rpm for 24 h at 37 °C. Overnight bacterial culture was centrifuged at 1500 xg for 5 min, supernatant was removed and cell pellet was suspended in 2 ml PBS.

#### 2.2.1 RAPD-PCR molecular typing of *K. pneumoniae* isolates

To assess the genetic diversity of the clinical isolates, Random Amplified Polymorphic DNA (RAPD)-PCR was performed. Genomic DNA was extracted from overnight bacterial cultures using a commercial bacterial genomic DNA extraction kit according to the manufacturer’s instructions. RAPD-PCR was carried out using four arbitrary primers: OPA-3 (5′-AGTCAGCCAC-3′), OPA-8 (5′-GTGACGTAGG-3′), and OPA-10 (5′-GTGATCGCAG-3′). Amplification reactions were performed in a final volume of 12.5 μL using GoTaq^®^ Green Master Mix (Promega Corporation, Madison, WI, USA). The thermal cycling program consisted of an initial denaturation step at 94 °C for 4 min, followed by 40 cycles of denaturation at 94 °C for 1 min, annealing at 34 °C for 1 min, and extension at 72 °C for 2 min. A final extension step was carried out at 72 °C for 10 min. The amplified products were resolved by electrophoresis on 1.5% agarose gels. RAPD banding patterns of K1, K2 and K3 generated by the different primers were compared among isolates to evaluate genetic diversity and distinguishability.

### 2.3 *K. pneumoniae* growth analysis in TSB and CSE-TSB

The optical density of overnight cultures of *K. pneumoniae* strains were measured and then diluted to obtain equivalent amounts to 4 × 10^4^ CFU in a total volume 200 µL of TSB or 5%, 10%, 20%, 25%, 50% or 75% CSE-TSB medium in a 96 well Costar plate. OD600 readings were taken every 1 hour for 7 h using a microplate reader (Epoch™ Microplate Spectrophotometer, BioTek, USA). Growth curves were graphed as the mean of triplicates (Vargas Buonfiglio et al., 2018; Lacoma et al., 2019).

### 2.4 Detection of virulence genes in *K. pneumoniae*

*K. pneumoniae* was inoculated into a MacConkey agar plate for overnight culture at 37 °C. Bacterial colonies were collected and incubated in broth and broth treated with 10% CSE for 2 h until the OD600 value = 0.6. Total RNA was extracted by using TRIzol™ Reagent (Thermo Scientific, USA) and cDNA synthesis was carried out using High Capacity cDNA Reverse Transcription Kit, Applied Biosystem, USA following manufacturer’s instructions (McEachern et al., 2015; Chien et al., 2020). qRT-PCR was used for determining the expression of the following virulence genes (*mrkA*, *luxS* and *AcrB*) (**Table 2**). Experiments were carried out using a 7500 Fast Real-Time PCR System (Applied Biosystems, Singapore) and the Maxima SYBR Green/ROX qPCR Master Mix (2X) (Thermo Scientific, USA). The threshold cycle (Ct) values were determined using 7500 software, version 2.3 (Applied Biosystems, Singapore). The 2^-Δct^ method was used to reflect the relative expression of mRNA molecules using 16S rRNA as a housekeeping gene.

**Table 2:** list of primers and conditions used for detection of the target genes.

Primer name	Description	Sequence (5`-3`)	Annealing	Reference
mrkA-F mrkA-R	Mannose-resistant Klebsiella	ACGTCTCTAACTGCCAGGC TAGCCCTGTTGTTTGCTGGT	60	(Vuotto et al., 2017)
luxS-F luxS-R	S-ribosylhomocysteine lyase	AGTGATGCCGGAACGCGG CGGTGTACCAATCAGGCTC	60	
AcrB-F AcrB-R	acriflavine resistance B	CAATACGGAAGAGTTTGGCA CAGACGAACCTGGGAACC	58	
16S rRNA-F 16S rRNA-R	16S ribosomal RNA	TGTCGTCAGCTCGTGTTGTG ATCCCCACCTTCCTCCAGTT	60	(Wang et al., 2019)

### 2.5 Biofilm formation assay

The effect of cigarette smoke extract (CSE) on biofilm formation by *K pneumoniae* clinical isolates was evaluated using the crystal violet staining method. Briefly, overnight cultures of the bacterial isolates (K1, K2, and K3) were grown in tryptic soy broth (TSB) at 37 °C with shaking. Cultures were adjusted to approximately 1 × 10^6^ CFU/mL in fresh TSB.

Aliquots (200 µL) of the standardized bacterial suspension were added to sterile, flat-bottom 96-well polystyrene microtiter plates in the presence or absence of 10% CSE. Each condition was tested in triplicate. Plates were incubated statically at 37 °C for 24 h to allow biofilm formation.

Following incubation, planktonic cells were removed, and wells were washed 3 times with sterile PBS to eliminate non-adherent bacteria. The attached biofilms were then fixed with methanol and stained with 0.1% (w/v) crystal violet solution at room temperature. Excess stain was removed by washing, and the plates were air-dried. The bound crystal violet was solubilized using 200 µL of 95% ethanol, and biofilm biomass was quantified by measuring the optical density at 570 nm (OD570) using a microplate reader (Epoch™, BioTek, USA).

Biofilm formation was expressed as mean OD570 values, and comparisons between groups were performed using the Mann-Whitney test. A p-value < 0.05 was considered statistically significant.

Background optical density from blank wells containing sterile medium was subtracted from all measurements to correct for non-specific staining.

### 2.6 Determination of minimum inhibitory concentration

The effect of cigarette smoke extract (CSE) on the antimicrobial susceptibility of *Klebsiella pneumoniae* clinical isolates was evaluated by determining the MIC using the broth microdilution method. Briefly, overnight cultures of the bacterial isolates (K1, K2, and K3) were grown in TSB at 37 °C with shaking and adjusted to approximately 5 × 10^5^ CFU/mL in Mueller–Hinton broth.

Serial two-fold dilutions of selected antibiotics, including ciprofloxacin, tigecycline, meropenem, and gentamicin, were prepared in sterile 96-well microtiter plates. Bacterial suspensions were added to each well in the presence or absence of 10% CSE to a final volume of 200 µL per well. Each condition was tested in triplicate.

The plates were incubated at 37 °C for 18–20 h under aerobic conditions. MIC values were defined as the lowest concentration of the antibiotic that completely inhibited visible bacterial growth. MIC values were interpreted according to CLSI guidelines. Differences in MIC values between CSE-treated and untreated groups were recorded and compared.

### 2.7 THP-1 cells (human monocytic leukemia cell line)

THP-1 was obtained from American Type Culture Collection-ATCC and maintained in RPMI 1640 medium supplemented with 10% (v/v) fetal bovine serum and 1% (v/v) penicillin - streptomycin (Gibco, USA). THP-1 cells 5 × 10^5^ were differentiated using 0.5 ng/ml phorbol 12-myristate 13-acetate (PMA, Sigma-Aldrich) for 48 h. THP-1 cell differentiation was enhanced by adding fresh media without PMA for a further 24 h. The morphological changes and adherence of the THP-1 cells were then observed under a light microscope (OLYMPUS, Japan) (Alqarni et al., 2019).

### 2.8 Gentamicin protection assay

THP-1 cells were exposed to CSE and then infected with *K. pneumoniae* for 3 hours following a protocol previously reported (Li et al., 2020), with some modifications. Briefly, freshly prepared 10% CSE extract was added to the cells and incubated for 3 hours. For the infection, the bacteria were added to the THP-1 macrophages at a multiplicity of infection (MOI) of 10. After 3 hours of contact at 37 °C in 5% CO_2_, the macrophages were washed three times with PBS to eliminate any extracellular bacteria and a medium containing 100 μl of 50 mg/ml gentamicin (Sigma-Aldrich, Germany) was added and incubated for 1 h at 37°C then treated with 1 mL 0.5% Triton X-100 (Sigma-Aldrich, Germany) in PBS for 20 min at 37 °C. Tenfold serial dilutions of cell lysates in broth were plated onto MacConkey Agar (Oxoid; Thermo Fisher Oxoid, UK), and incubated overnight at 37 °C to count the intracellular bacteria (Colony-Forming Units, CFU).

### 2.9 Phagocytosis assay by flow cytometry

The impact of cigarette smoke extract (CSE) on neutrophil phagocytosis of *K pneumoniae* was evaluated using flow cytometry. Briefly, *K. pneumoniae* was cultured to mid-logarithmic phase (OD600 = 0.4–0.6), harvested by centrifugation, washed with PBS, and fluorescently labeled with fluorescein isothiocyanate (FITC) (0.1 mg/mL; Sigma-Aldrich, Germany) as previously described (Pereira et al., 2021). Excess dye was removed by washing three times with PBS.

For opsonization, 200 μL of FITC-labeled *K. pneumoniae* (4 × 10^7^ cells) were incubated with 100 μL of pooled normal human serum (10% v/v) at 37 °C for 20 min. Neutrophils were isolated from peripheral blood using standard density gradient centrifugation and resuspended in RPMI 1640 medium. Cells were adjusted to a concentration of 1 × 10^6^ cells/mL. Opsonized bacteria were then co-incubated with neutrophils at a multiplicity of infection (MOI) of 10 in the presence or absence of 10% CSE. Incubations were performed at 37 °C in 5% CO_2_ for different time points (10, 30, and 60 min). Following incubation, cells were washed with cold PBS to remove non-adherent bacteria. To distinguish internalized from extracellular bacteria, trypan blue (0.4%) was added immediately prior to analysis to quench extracellular fluorescence.

Phagocytosis was quantified by flow cytometry using a BD FACSCalibur (BD Biosciences, USA). Data were analyzed to determine the percentage of FITC-positive neutrophils, representing the extent of bacterial uptake per cell. All experiments were performed in triplicate. Statistical comparisons between groups were carried out using the Mann-Whitney test, with p < 0.05 considered statistically significant.

### 2.10 Cytokine gene expression analysis in THP-1 macrophages

The effect of cigarette smoke extract (CSE) on cytokine responses during *Klebsiella pneumoniae* infection was evaluated in THP-1-derived macrophages using quantitative real-time PCR (qRT-PCR). THP -1 (1 x 10^6^ macrophage cells/ml) were suspended in RPMI 1640 medium (Gibco, USA) in the presence of 10% CSE. Then, THP-1 cells were challenged with *K. pneumoniae* (MOI = 10) and then harvested at 2, 4, and 6 h post-infection to evaluate cytokine gene expression. Control cells without CSE were included that were incubated in normal RPMI without CSE. Cells were subjected to RNA extraction and cDNA synthesis (High-Capacity cDNA Reverse Transcription Kit, Applied Biosystem, USA) for quantitative expression of cytokines. The expression of interleukin-1β (IL-1β) and tumor necrosis factor alpha (TNF-α) have proinflammatory function and anti-inflammatory cytokines: interleukin-10 (IL-10). Experiments were carried out using the 7500 Fast Real-Time PCR System (Applied Biosystems, Singapore) and the Maxima SYBR Green Master Mix (Thermo Scientific, USA). GAPDH was used as a reference gene. Relative gene expression was calculated using the 2^-^ΔCt method. Fold change was determined as the ratio of gene expression in CSE-treated cells relative to control cells at each time point. Amplification protocol involved an initial denaturation of 95 °C for 10 min, followed by 40 cycles at 95°C for 15 sec and 60 °C for 30 sec (Mortaz et al., 2015; Ak et al., 2016). All experiments were performed in triplicate. Statistical comparisons between groups were conducted using the Mann-Whitney test.

## 3 Results

### 3.1 Antimicrobial susceptibility profile of *K. pneumoniae* clinical isolates

The antimicrobial susceptibility profiles of the *K. pneumoniae* clinical isolates (K1, K2, and K3) are summarized in **Table 1**. All isolates exhibited a multidrug-resistant (MDR) phenotype and were consistent with extended-spectrum β-lactamase (ESBL) production, as evidenced by resistance to third- and fourth-generation cephalosporins (ceftazidime, ceftriaxone, and cefepime) and β-lactam/β-lactamase inhibitor combinations.

In addition, the isolates demonstrated resistance to multiple non-β-lactam antibiotics, including fluoroquinolones and trimethoprim-sulfamethoxazole. Variable susceptibility was observed among aminoglycosides and nitrofurantoin, indicating heterogeneity among the isolates.

Notably, all isolates remained susceptible to carbapenems (imipenem and meropenem), as well as colistin and tigecycline. These findings confirm that the selected isolates represent different clinically relevant MDR *K. pneumoniae* strains with preserved susceptibility to last-line therapeutic agents. RAPD-PCR fingerprinting revealed distinct banding patterns among isolates K1, K2, and K3 (**Supplementary Figure 1**), indicating genetic heterogeneity and confirming that the isolates were genetically distinguishable.

### 3.2 High concentrations of CSE decreases bacterial growth

**Figure 1:**
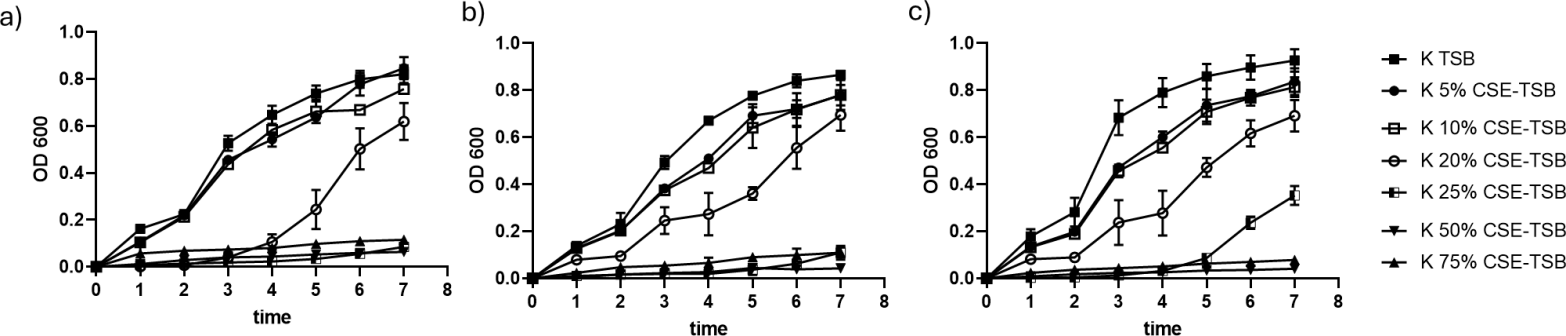
Growth dynamics of three *K. clinical isolates* in the presence of increasing concentrations of CSE. Bacterial cultures (4 × 10^4^ CFU) were grown in tryptic soy broth (TSB) supplemented with 0%, 5%, 10%, 20%, 25%, 50%, or 75% CSE. Growth was monitored by measuring optical density at 600 nm (OD600) every hour for 7 hours at 37 °C. Panels show **(A)** *K. pneumoniae* isolate 1, **(B)** isolate 2, and **(C)** isolate 3. Data represent the mean of triplicate wells.

To test the effect of CSE exposure on the bacterial growth rate, we determined the growth rate of the bacteria in presence of a range of CSE concentrations (5%, 10%, 20%, 25%, 50% and 75%). **Figure 1** illustrates the growth dynamics of three *Klebsiella pneumoniae* clinical isolates exposed to increasing concentrations of CSE over a 7-hour period. Across all three isolates (A, B, and C), there is a consistent dose-dependent inhibitory effect of CSE on bacterial growth. At lower concentrations (5% and 10%), the growth curves are non-significantly reduced compared to the control (0% CSE), suggesting that minimal exposure already imposes mild stress on the bacterial growth rate. This is reflected by a modest delay in reaching the exponential phase. As CSE concentrations increase to 25%, 50%, and 75%, a marked suppression of growth is observed in all strains, characterized by a significant lag in bacterial replication and greatly reduced overall biomass. Particularly at 75% CSE, growth is almost completely inhibited.

### 3.3 Cigarette smoke increases *K. pneumoniae* expression of virulence- and resistance-associated genes

**Figure 2:**
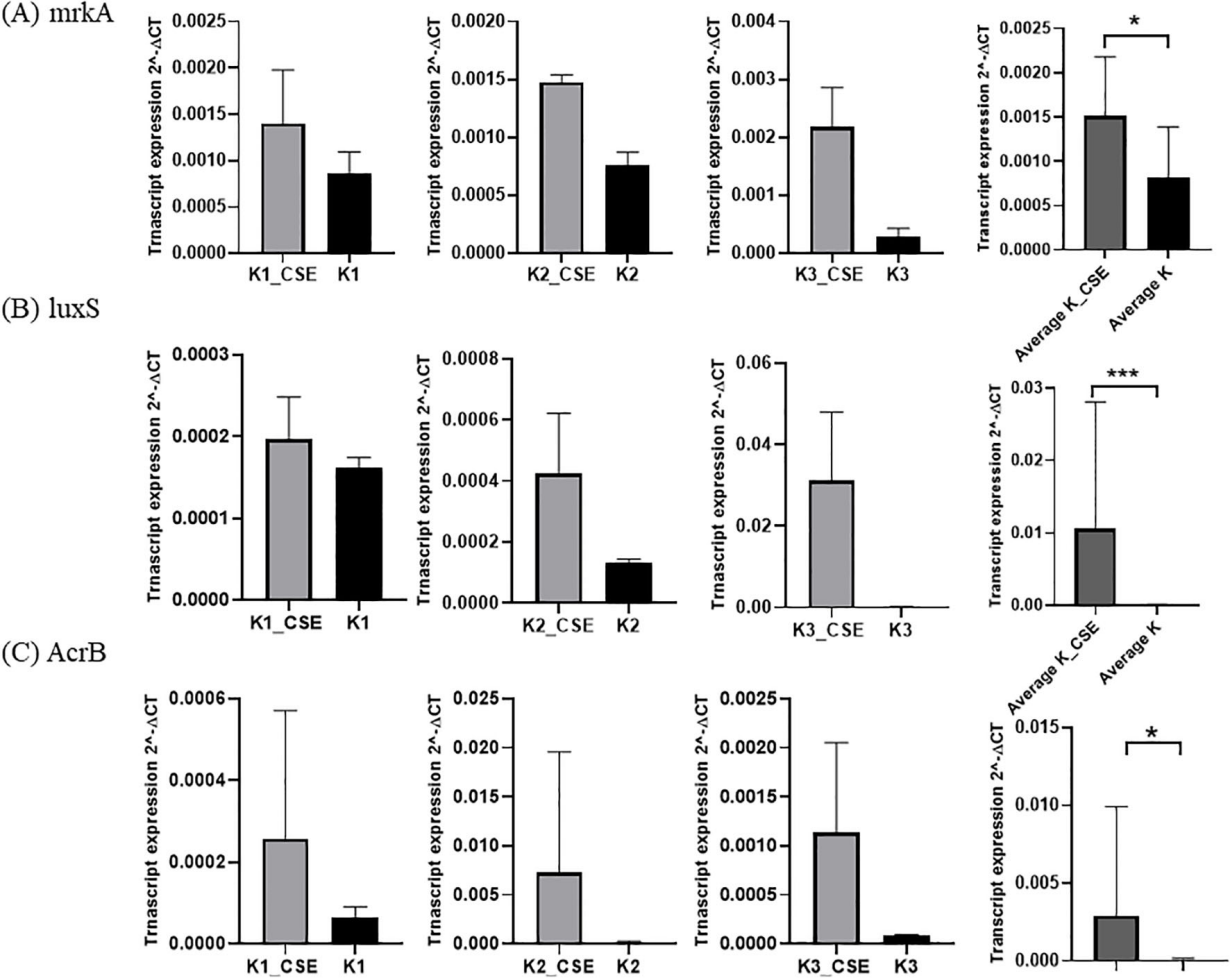
Effect of CSE on the expression of virulence and resistance-associated genes. Transcript levels of **(A)** *mrkA*, **(B)** *luxS*, and **(C)** *AcrB* were measured by qRT-PCR in 3 clinical isolates of *K. pneumoniae* (K1, K2, and K3) after 2-hour exposure to 10% CSE. Expression levels were normalized to 16S rRNA and calculated using the 2^–ΔCt method. Each gene panel shows individual strain responses and the averaged expression across all strains. Experiments were performed triplicates. Comparison between groups was carried out using the Mann-Whitney test, * denotes significant difference with p values < 0.05.

To evaluate the impact of CSE on the expression of virulence and resistance-related genes, qRT-PCR was performed on *K. pneumoniae* isolates exposed to 10% CSE for 2 hours. As shown in **Figure 2**, exposure to CSE led to a notable upregulation of *mrkA*, *luxS*, and *AcrB* transcripts across all three clinical isolates compared to untreated controls (*p* < 0.05). These findings suggest that cigarette smoke promotes enhanced expression of genes associated with biofilm formation (*mrkA*, *luxS*) and multidrug resistance (*AcrB*), potentially contributing to the increased virulence and persistence of *K. pneumoniae* under smoke-exposed conditions.

### 3.4 CSE enhances biofilm formation in *K pneumoniae*

To determine whether the observed upregulation of biofilm-associated genes (mrkA and luxS) translated into a functional phenotype, biofilm formation was assessed using a crystal violet assay. As shown in **Table 3**, exposure to 10% cigarette smoke extract (CSE) resulted in a significant increase in biofilm biomass across all three clinical isolates compared to untreated controls.

**Table 3:** Effect of CSE on biofilm biomass of *K. pneumoniae* isolates.

Isolate	Control (OD570) Mean ± SD	+10% CSE (OD570) Mean ± SD	% Change	P-value
K1	0.48 ± 0.05	0.58 ± 0.05	+20.8%	0.018
K2	0.52 ± 0.06	0.64 ± 0.06	+23.1%	0.012
K3	0.46 ± 0.04	0.63 ± 0.05	+37.0%	0.004

Biofilm formation was quantified using crystal violet staining and expressed as optical density at 570 nm (OD570). Data represent mean ± SD of three independent experiments. Statistical significance was determined using the Mann-Whitney test.

CSE exposure resulted in a consistent and significant increase in biofilm formation across all tested isolates, with biofilm biomass increasing by approximately 20.8-37% compared to controls (p < 0.05). These findings indicate that CSE promotes biofilm formation in *K. pneumoniae*, consistent with the observed upregulation of biofilm-associated genes.

### 3.5 CSE selectively alters antimicrobial susceptibility in *Klebsiella pneumoniae*

To determine whether the upregulation of the resistance-associated gene *AcrB* was associated with phenotypic changes in antimicrobial susceptibility, minimum inhibitory concentrations (MICs) of selected antibiotics were evaluated in the presence and absence of cigarette smoke extract (CSE). As shown in **Table 4**, exposure to 10% CSE resulted in a consistent two-fold increase in MIC values for ciprofloxacin and tigecycline across all tested isolates.

**Table 4:** Effect of cigarette smoke extract on MICs of selected antibiotics against *Klebsiella pneumoniae* isolates.

Antibiotic	Isolate	MIC (µg/mL) Control	MIC (µg/mL) +10% CSE	Fold Change
Ciprofloxacin	K1	2	4	2×
	K2	0.25	0.5	2×
	K3	2	4	2×
Tigecycline	K1	0.5	1	2×
	K2	0.5	1	2×
	K3	1	2	2×
Meropenem	K1	0.25	0.25	1× (no change)
	K2	0.25	0.25	1× (no change)
	K3	0.5	0.5	1× (no change)
Gentamicin	K1	2	2	1× (no change)
	K2	16	16	1× (no change)
	K3	16	16	1× (no change)

For ciprofloxacin, isolates K1 and K3, which were phenotypically resistant, exhibited further increases in MIC values (2 to 4 µg/mL), while K2 demonstrated a shift from susceptible to intermediate (0.25 to 0.5 µg/mL). Similarly, tigecycline MICs increased two-fold in all isolates (0.5–1 to 1–2 µg/mL), although values remained within the susceptible range.

In contrast, no changes in MIC values were observed for meropenem or gentamicin following CSE exposure, regardless of baseline susceptibility profiles.

These findings suggest that CSE may induce antimicrobial tolerance, selectively affecting antibiotics associated with efflux pump activity, while having minimal impact on non-substrate antibiotics.

### 3.6 CSE-mediated increased *K. pneumoniae* invasion of THP-1 cells.

**Figure 3:**
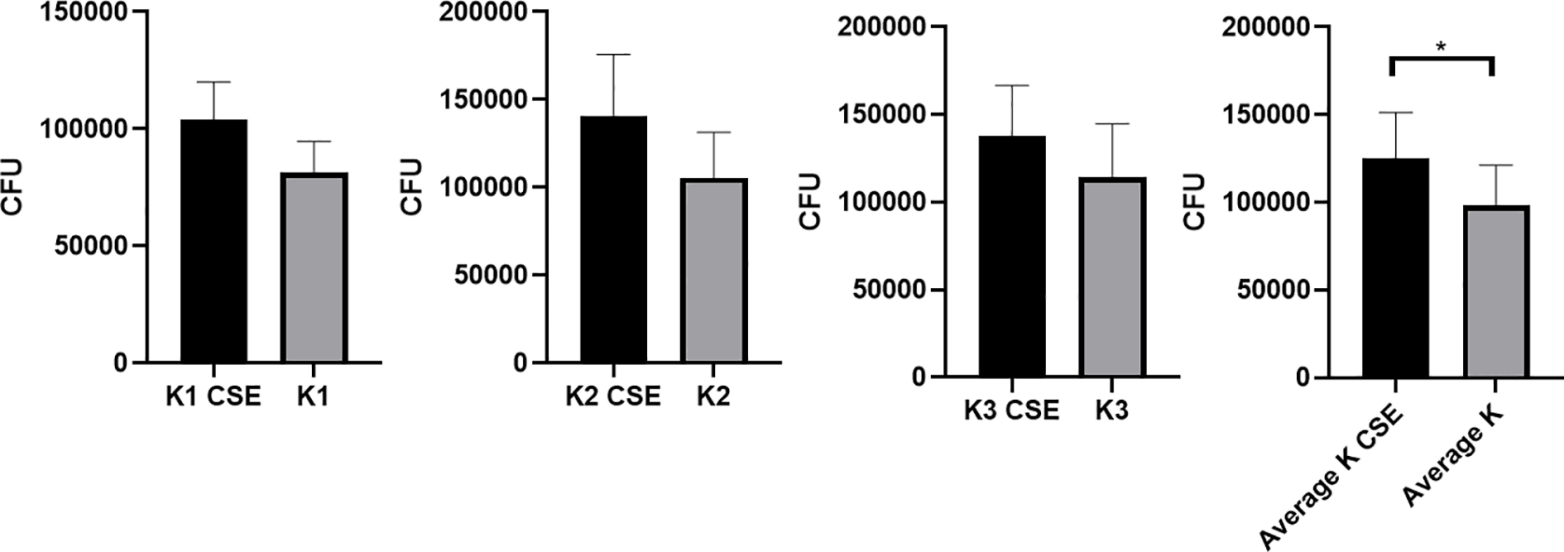
CSE increased the ability of Klebsiella to invade macrophages. THP-1 cells were treated with 10% CSE or control medium without CSE. Cells were then subjected to *K. pneumoniae* infection followed by gentamycin treatment. Cells were lysed, the cell lysates were diluted to inoculate TSB plates and colonies were counted. Graph represents mean ± SD of three independent experiments. Comparison between groups was carried out using the Mann-Whitney test, * denotes significant difference with p values < 0.05.

To assess whether CSE influences bacterial invasion of macrophages, the intracellular invasion of CSE-treated THP-1 cells by *K. pneumoniae* was quantified by the gentamicin protection assay. As shown in **Figure 3**, when Klebsiella were cocultured with THP-1 cells and exposed to 10% CSE, their ability to invade the THP-1 cells were increased. The number of intracellular *K. pneumoniae* was significantly higher compared to macrophages infected with *K. pneumoniae* in normal media. These findings suggest that CSE enhances the invasive capacity of *K. pneumoniae*, potentially impairing the phagocytic defense of macrophages and facilitating bacterial persistence within host cells.

### 3.7 CSE impairs neutrophil phagocytic activity in a time-dependent manner

**Figure 4:**
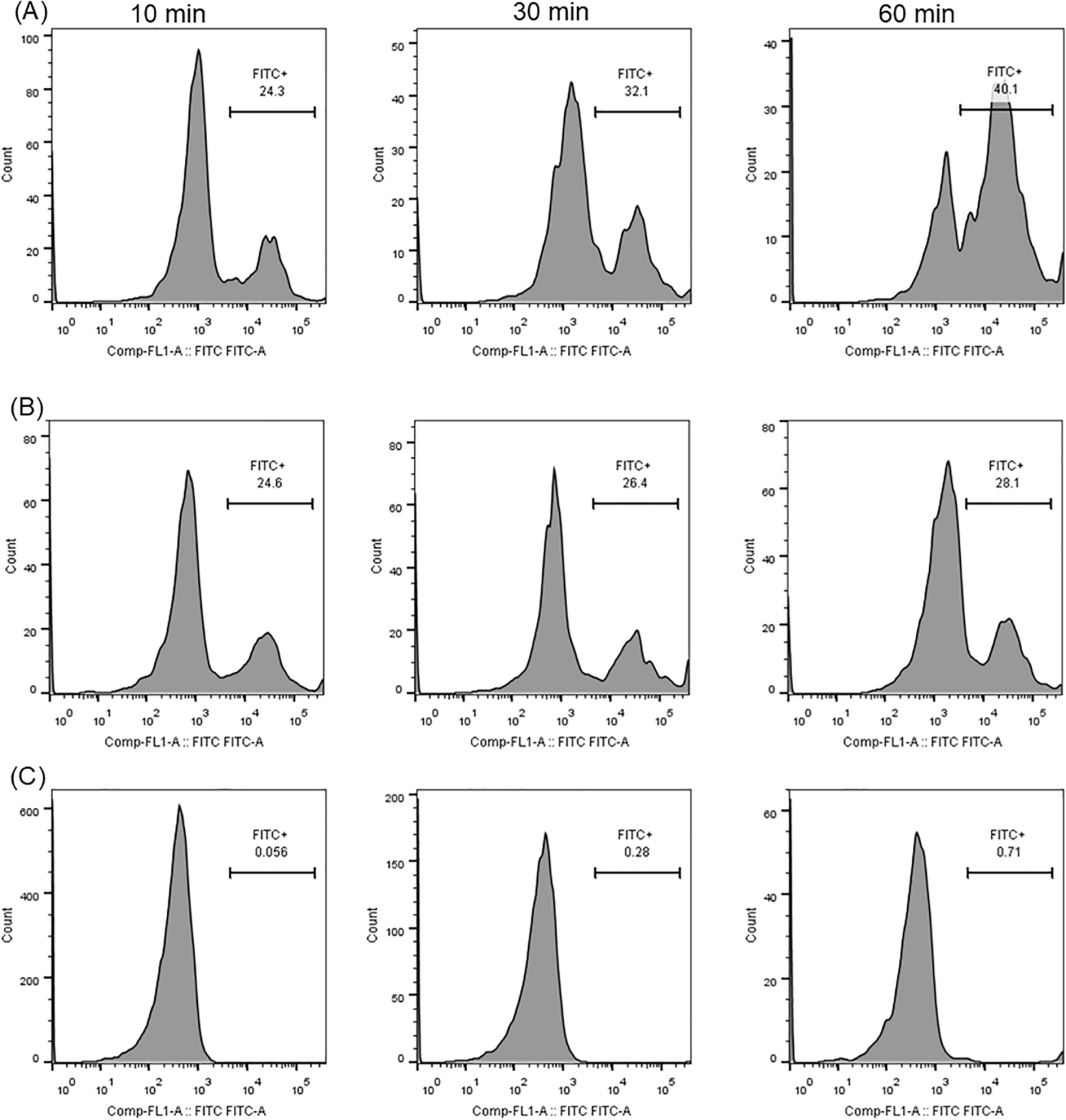
Effect of CSE on neutrophil phagocytose bacteria. Neutrophil cells (1x10^6^) were incubated with FITC-labelled *K. pneumoniae* in presence and absence of CSE. Exposure to CSE decreases the ability of neutrophils to engulf bacteria. **(A)** Neutrophils and Bacteria (without CSE). **(B)** Neutrophils and Bacteria (with CSE). **(C)** Neutrophils (control).

To evaluate the effect of cigarette smoke extract (CSE) on neutrophil phagocytic function, uptake of FITC-labeled *K. pneumoniae* was quantified by flow cytometry at different time points. As shown in **Figure 4**, CSE exposure had minimal effect on early phagocytosis at 10 min, with comparable percentages of FITC-positive neutrophils observed between control and CSE-treated groups (24.3% vs. 24.6%).

However, at later time points, a clear reduction in phagocytic activity was observed in CSE-treated neutrophils. At 30 min, the percentage of FITC-positive cells decreased from 32.1% in controls to 26.4% following CSE exposure (−17.8%), and at 60 min, a more pronounced reduction was observed (40.1% vs. 28.1%, −29.9%).

These findings indicate that CSE impairs neutrophil phagocytic capacity in a time-dependent manner, with the inhibitory effect becoming more evident during sustained exposure. This suggests that cigarette smoke may compromise innate immune function by limiting the ability of neutrophils to effectively internalize bacterial pathogens.

### 3.8 Cigarette smoke enhances proinflammatory cytokine expression post *K. pneumoniae* challenge

**Figure 5:**
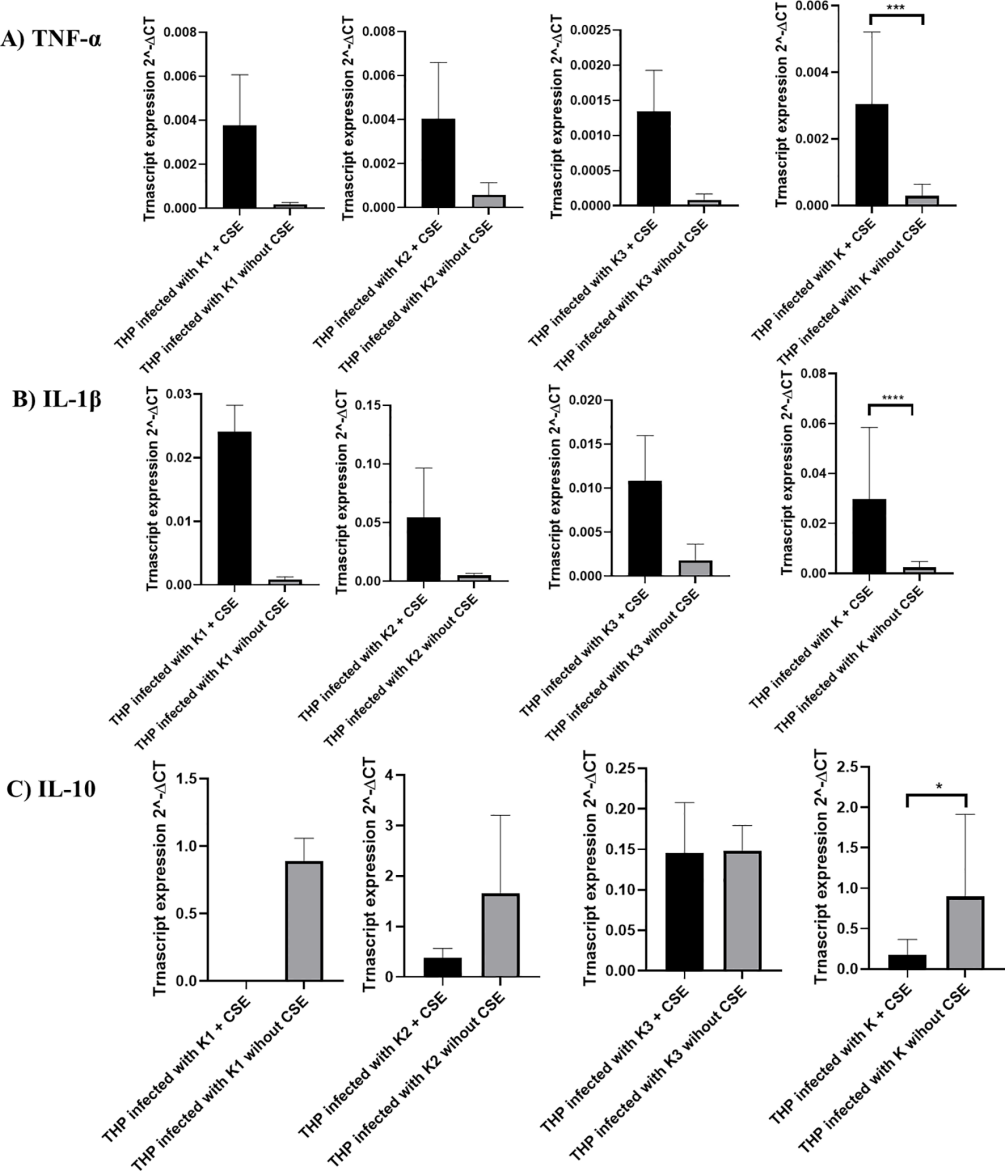
Effect of CSE on cytokine gene expression in *K. pneumoniae-*challenged THP-1 cells. THP-1 cells were incubated with *K. pneumoniae* in the presence or absence of 10% CSE and the transcript levels of the proinflammatory cytokines TNF-α **(A)**, IL-1β **(B)**, and the anti-inflammatory cytokine IL-10 **(C)**, were assessed after 2 h by qRT-PCR and normalized to GAPDH. Data are presented as mean ± SD of three independent experiments. P values were calculated using Mann-Whitney test. *p < 0.05; ***p < 0.001; ****p < 0.0001.

To assess the effect of CSE on cytokine responses during bacterial infection, the expression of pro- and anti-inflammatory cytokines was evaluated in *K. pneumoniae*-infected THP-1 macrophages using qRT-PCR. As shown in **Figure 5**, at 2 h post-infection, CSE exposure significantly increased the expression of TNF-α (p = 0.0002) and IL-1β (p < 0.0001), while significantly reducing the expression of the anti-inflammatory cytokine IL-10 (p = 0.0188) compared to controls.

To further support these findings, cytokine expression was also analyzed at additional time points (4 and 6 h post-infection). Consistent with the 2 h results, CSE-treated cells exhibited sustained upregulation of TNF-α and IL-1β and persistent suppression of IL-10 at all examined time points. A summary of these data is presented in **Table 5**. Collectively, these results confirm that CSE promotes a pro-inflammatory and dysregulated immune response during *K. pneumoniae* infection.

**Table 5:** Summary of the effect of CSE on cytokine gene expression in *K. pneumoniae*-infected THP-1 macrophages.

Cytokine	Time (h)	Control (2^-^ΔCt)	+10% CSE (2^-^ΔCt)	Fold Change	P-value
TNF-α	2	0.00029	0.00304	10.5 ↑	0.0002
	4	0.00040	0.00560	14.0 ↑	<0.01
	6	0.00050	0.00400	8.0 ↑	<0.01
IL-1β	2	0.00252	0.02969	11.8 ↑	<0.0001
	4	0.00450	0.06000	13.3 ↑	<0.01
	6	0.00750	0.11000	14.7 ↑	<0.01
IL-10	2	0.897	0.174	0.19 ↓	0.0188
	4	1.80	0.60	0.33 ↓	<0.01
	6	3.20	1.10	0.34 ↓	<0.01

Gene expression was calculated using the 2^-^ΔCt method (normalized to GAPDH). Fold change represents the ratio of CSE-treated to control groups. Data are shown as mean ± SD (n = 3). Statistical significance was determined using the Mann–Whitney test (p < 0.05).

## 4 Discussion

This study demonstrates that cigarette smoke extract (CSE) significantly modulates the virulence profile of *K. pneumoniae* and alters host immune responses. Specifically, we observed that although CSE reduced bacterial growth at higher concentrations, it paradoxically increased expression of virulence-related genes, enhanced bacterial invasion into macrophages, and induced a proinflammatory cytokine profile. These findings were further supported by functional assays, including increased biofilm formation, selective changes in antimicrobial susceptibility, impaired neutrophil phagocytosis, and consistent cytokine responses across multiple time points. Collectively, these results underscore the complex and potentially deleterious effects of cigarette smoke on host–pathogen dynamics and provide mechanistic insights into the increased susceptibility to bacterial infections among smokers.

Our results demonstrated that cigarette smoke (CS) reduced the growth rate of *Klebsiella pneumoniae* only at higher concentrations. This finding aligns with previous studies reporting that Gram-positive cocci are more susceptible to CS, exhibiting growth inhibition at lower concentrations compared to Gram-negative rods (Ertel et al., 1991).

This study demonstrates that exposure to CSE significantly enhances the expression of key virulence- and resistance-associated genes in *K pneumoniae*. Specifically, we observed that genes involved in biofilm formation (*mrkA* and *luxS*) and multidrug resistance (*AcrB*) were upregulated in response to 10% CSE, indicating that cigarette smoke may directly influence the pathogenic potential of *K. pneumoniae*. The combination of CS and *K pneumoniae* causes earlier onset, more severe, and longer-lasting airway epithelial barrier damage than either alone, likely through activation of the EGFR/ERK1/2 signaling pathway (Li et al., 2023).

The *mrkA* gene encodes the major subunit of type 3 fimbriae, which plays a crucial role in bacterial adhesion and biofilm formation, an essential factor in chronic colonization and immune evasion. Our results showed that *mrkA* expression increased following CSE exposure, suggesting that smoke components may enhance the ability of *K. pneumoniae* to adhere to host surfaces and resist clearance mechanisms. This finding is consistent with prior work demonstrating increased biofilm production in smoke-exposed respiratory pathogens (Brown, 2021; Li and Ni, 2023).

Similarly, the *luxS* gene, which is part of the quorum-sensing pathway regulating biofilm and virulence factor expression, was markedly upregulated. It encodes S-ribosylhomocysteine lyase that facilitate biofilm formation The sharp increase in *luxS* expression suggests that cigarette smoke may serve as an environmental signal, triggering collective bacterial behavior conducive to persistence and virulence (Chen et al., 2020). Importantly, this transcriptional upregulation was supported by functional validation, as CSE exposure significantly increased biofilm formation across all tested clinical isolates. Given that biofilm development is a key virulence-associated trait in *K. pneumoniae*, these findings indicate that CSE promotes a more persistent bacterial phenotype.

In addition to virulence factors, we observed upregulation of the *AcrB* efflux pump gene, which is known to mediate resistance to a broad range of antibiotics. This finding highlights a potential mechanism by which *K. pneumoniae* might become more resistant to antimicrobial treatment following smoke exposure. Upregulation of this efflux protein is one of the important mechanisms in multidrug-resistant *K. pneumoniae* isolates may represent a survival strategy enabling the pathogen to withstand both host defenses and therapeutic agents (Vareschi et al., 2025).

Furthermore, CSE exposure resulted in selective changes in antimicrobial susceptibility, with increased MIC values observed for ciprofloxacin and tigecycline, while no changes were detected for meropenem or gentamicin. This selective pattern is consistent with efflux-mediated resistance mechanisms and aligns with previous studies demonstrating that environmental stressors can induce multidrug efflux systems in Gram-negative bacteria (Nikaido, 2009; Li et al., 2015). The AcrAB-TolC efflux pump, in particular, has been shown to contribute to reduced susceptibility to fluoroquinolones and tetracycline-class antibiotics, including tigecycline (Ardehali et al., 2019). Moreover, exposure to cigarette smoke has been reported to enhance bacterial virulence and stress responses, which may further promote adaptive resistance phenotypes (Bagaitkar et al., 2008). Together, these findings support the functional relevance of *AcrB* upregulation and suggest that CSE exposure may enhance efflux-associated antimicrobial tolerance.

These findings suggest that cigarette smoke not only alters the host environment but also directly stimulates bacterial adaptations that enhance survival, persistence, and pathogenicity. This aligns with epidemiological evidence linking smoking to increased risk, severity and mortality of respiratory infections, particularly those caused by Gram-negative pathogens such as *K. pneumoniae* (Feldman and Anderson, 2025; Joshy et al., 2025).

Cigarette smoking is the most significant risk factor for developing invasive pneumococcal disease in immunocompetent adults (Nuorti et al., 2000). It disrupts multiple functions of alveolar macrophages by impairing their recruitment, phenotype, and immune and homeostatic activities, contributing to lung disease and increased susceptibility to infection (Lugg et al., 2022). Alveolar macrophage phagocytic function is significantly reduced in COPD patients who smoke compared to those who have quit smoking (Dewhurst et al., 2017).

In addition, CSE exposure significantly impaired neutrophil phagocytic activity, indicating a direct inhibitory effect on innate immune function. Neutrophils represent a primary line of defense against *K. pneumoniae*, and their ability to effectively phagocytose bacteria is critical for infection control. Previous studies have shown that cigarette smoke exposure compromises neutrophil function, including reduced phagocytosis and impaired bacterial clearance (Phipps et al., 2010; Hodge et al., 2011). Therefore, the observed reduction in phagocytic activity may contribute to enhanced bacterial persistence and survival despite an activated inflammatory response.

Cigarette smoke extract impairs the phagocytic ability of alveolar macrophages against non-typeable *Haemophilus influenzae*, a common upper respiratory tract colonizer, potentially by weakening phosphoinositide 3-kinase signaling pathways activated by Gram-negative bacteria (Marti-Lliteras et al., 2009). In addition, cigarette smoke induced modification of extracellular matrix proteins impairs macrophage phagocytosis of apoptotic cells by redirecting receptor activity toward carbonyl-adduct recognition, leading to increased macrophage adhesion and reduced clearance of inflammation (Kirkham et al., 2004).

TLR4, a surface receptor that detects lipopolysaccharide (LPS) from Gram-negative bacteria, shows decreased expression in monocyte-derived macrophages after exposure to cigarette smoke (Sarir et al., 2009). These findings align with earlier reports showing that cigarette smoke impaired production of chemokines, migratory potential, and suppression of bacterial phagocytosis by macrophages (Liao et al., 2025).

Consistent with these observations, we found that the intracellular invasion of *K. pneumoniae* was significantly increased in CSE-treated THP-1 macrophages, highlighting the ability of cigarette smoke to impair innate immune defenses and promote bacterial persistence. Our findings reveal that CSE significantly skews the cytokine response of THP-1 macrophages during *K. pneumoniae* infection, amplifying proinflammatory mediators (TNF-α, IL-1β) while suppressing the anti-inflammatory cytokine IL-10, and this effect was consistently observed across multiple time points, indicating a sustained dysregulated inflammatory response. This inflammatory imbalance is consistent with prior data showing that CSE increases macrophage IL-8 expression and through the generation of reactive oxygen species and activation of NF-κB. Additionally, CS significantly amplifies IL-8 production when combined with TNF-α (Sarir et al., 2010).

Our results are consistent with findings in other cell types. For example, exposure of human gingival epithelial cells to cigarette smoke increases the expression of various β-defensins and proinflammatory cytokines (Semlali et al., 2012). Similarly, CSE-stimulated BEAS-2B airway epithelial cells exhibit elevated IL-1β production in a dose-dependent manner Pereira, Oliveira (Pereira et al., 2021) Notably, in the presence of *Cryptococcus neoformans*, CSE induces an anti-inflammatory effect in bronchial epithelial cells, potentially facilitating fungal infection. In another study, CSE exposure (1%) synergistically enhanced TNF-α gene expression in human airway epithelial NCI-H292 cells (Baginski et al., 2006).

The central role of TNF-α in cigarette smoke induced inflammation is well-established. Cigarette smoke increases TNF-α expression, which contributes to acute inflammatory responses and connective tissue degradation (Churg et al., 2002). In murine models, TNF-α accounts for approximately 70% of cigarette smoke–induced emphysema development, underscoring its importance in disease progression (Churg et al., 2004).

However, cigarette smoke also exerts immunosuppressive effects. Knobloch and colleagues reported that cytokine responses to bacterial stimuli are impaired in smokers and COPD patients, potentially due to reduced NOD2 and TLR4 expression and/or increased IL-10 production. Knobloch, Panek (Knobloch et al., 2019). Additionally, cigarette smoke stimulates IL-8 release from human airway smooth muscle cells, an effect intensified by elevated TNF-α levels commonly observed in COPD (Oltmanns et al., 2005).

On a molecular level, cigarette smoke reduces the activity and expression of SIRT1-a key member of the silent information regulator 2 (Sir2) family-in immune cells. This reduction leads to enhanced NF-κB activation and increased proinflammatory cytokine release. Disruption of the SIRT1-NF-κB interaction further contributes to sustained inflammation and may play a role in smoke-induced lung aging (Yang et al., 2007).

While the present study provides important mechanistic insights into the effects of CSE on *K. pneumoniae* and host-pathogen interactions, certain limitations should be considered. The findings are based on *in vitro* models, and further studies incorporating *in vivo* systems would help extend these observations. Although RAPD-PCR confirmed the genetic heterogeneity of the clinical isolates, higher-resolution molecular typing approaches, such as multilocus sequence typing (MLST) or whole-genome sequencing, may provide additional insights into strain-specific characteristics and epidemiological relationships. Future investigations addressing these aspects will further strengthen our understanding of CSE-mediated effects on bacterial infections.

## 5 Conclusion

This study demonstrates that cigarette smoke extract (CSE) significantly alters both bacterial and host immune responses in the context of *Klebsiella pneumoniae* infection. While CSE inhibited bacterial growth at higher concentrations, it paradoxically upregulated key virulence and antibiotic resistance genes (*mrkA*, *luxS*, *AcrB*) and enhanced bacterial invasion into macrophages. Simultaneously, CSE-exposed THP-1 macrophages exhibited a dysregulated cytokine profile characterized by elevated proinflammatory mediators (TNF-α, IL-1β) and suppressed anti-inflammatory signaling (IL-10). These findings suggest a harmful synergy between cigarette smoke and pathogenic bacteria, in which CSE not only compromises immune defense but also promotes more aggressive bacterial behavior. The dual impact of cigarette smoke on both pathogen and host provides critical insight into the heightened risk and severity of respiratory infections among smokers.

## Data Availability

The original contributions presented in the study are included in the article/**Supplementary Material**. Further inquiries can be directed to the corresponding author.
