# Supplementary material for: Cigarette smoke modulates virulence-related properties and host immune responses in Klebsiella pneumoniae
Source: Front Cell Infect Microbiol. 2026 Jul 23;16:1787424. doi: 10.3389/fcimb.2026.1787424 (PMC13442400; doi:10.3389/fcimb.2026.1787424)
Supplement: Supplementary file 1 [file Table1.docx]

**Supplementary Figure S1.** RAPD-PCR fingerprinting of the three clinical *Klebsiella pneumoniae* isolates using primers OPA-3 (A), OPA-10 (B), and OPA-8 (C). Lane M, 1.5 kb DNA ladder; lanes K1–K3, clinical isolates. Distinct RAPD profiles were observed among the isolates, indicating genetic heterogeneity.
